# The importance of regulatory data protection or exclusive use and other forms of intellectual property rights in the crop protection industry†

**DOI:** 10.1002/ps.4316

**Published:** 2016-06-17

**Authors:** Michael J Carroll

**Affiliations:** ^1^ Arysta Life ScienceSloughBerkshireUK

**Keywords:** registration, regulatory data protection exclusive use period, compensation, data call‐in

## Abstract

In order for a chemical plant protection product to be authorised for sale a registration dossier has to be assembled to demonstrate safety and efficacy to the satisfaction of government regulators. These studies and tests are protected for a period of 10 years in Europe, North America and some other jurisdictions from the date of first product authorisation so that only the data owner can gain commercial benefit from the data. Subsequent regulatory reviews which require new studies should not result in further periods of regulatory data protection exclusive use for the new data but compensation should be payable to the data generator. © 2016 The Authors. *Pest Management Science* published by John Wiley & Sons Ltd on behalf of Society of Chemical Industry.

## 1 SUMMARY

In certain regulatory jurisdictions, regulatory data protection or exclusive use is granted to safety and efficacy data in the registration dossier necessary for marketing authorisation of chemical plant protection products. This time‐limited intellectual property right gives the data owner a period of usually 10 years in Europe and North America whereby no other company can use the data for commercial gain. Generating the safety and efficacy data for a chemical plant protection product is a multimillion dollar investment that is not guaranteed to be successful, and the investment continues after first authorisation with various regulatory reviews. Regulatory data protection or exclusive use therefore protects the investment in demonstrating the safety and efficacy of a chemical plant protection product. Subsequent regulatory reviews should not result in further periods of regulatory data protection exclusive use, but compensation should be payable to the data generator.

## 2 INTRODUCTION

In order for a chemical plant protection product to be authorised for sale, a registration dossier has to be assembled to demonstrate safety and efficacy to the satisfaction of government regulators. The registration dossier contains many components that are also covered by various forms of intellectual property right.

The patent usually covers the invention, which can be the active substance and the formulated products but also in some cases the manufacturing process, and these are described briefly in the registration dossier. Patents involve disclosure and annual payment by country and are time limited. In Europe and North America, patents for chemical plant protection products are typically of 20 years duration from filing, and in Europe they can also be extended by up to a further 5 years if the registration process delays market entry.

Trade secrets are found in the confidential business information section of the registration dossier and are typically the manufacturing process if not patented, the technical specification of the active ingredient and formulated product and any specific analytical methods that could betray the manufacturing process by extrapolation from individual impurities. Trade secrets are free but are only valid as long as they are kept secret, and there is always a dynamic conflict between whether to patent or keep as a trade secret any novel invention.

The trademark is used to protect the brand name of the formulated product, and there is now a current vogue for branding the active ingredient. These are similar to patents in that annual fees are paid by country to maintain the protection.

Copyright protects the chemical plant protection product label and, like trade secrets, is free, but there is an enhanced system in the United States that involves an initial one‐off payment.

Finally, there is regulatory data protection, or exclusive use as it is known in the United States, which protects the studies and tests that are undertaken to demonstrate safety and efficacy of the chemical plant protection product. For a global new product, these studies and tests can cost upwards of $US 350 million, and the costs are rising each year as new studies and tests are added to the list of regulatory requirements. These studies and tests are protected for a period of 10 years in Europe, North America and some other jurisdictions from the date of first product authorisation, so that no other company other than the originator can gain commercial benefit from the data. Regulatory data protection is free, but it is not given by every country. Some give 10 years, but others give nothing, and some give between 0 and 10 years as this particular intellectual property right is in the gift of the national jurisdiction.

## 3 THE FOUR STAGES IN THE DEVELOPMENT OF AGROCHEMICALS

It is important to be clear about intellectual property in that it is very much dependent on your economic perspective. Developing countries have little interest in intellectual property as they must pay for expensive foreign proprietary technology.^1^ On the other hand, developed countries with massive commercial investments in developing new technology wish to promote intellectual property rights to protect massive investments that initially have no guarantee of commercial success. The four stages in the development of agrochemicals are described in Table 1, and countries change their position on intellectual property as they pass through the different stages. Thus, intellectual property is not a moral issue; there is no absolute position that can be taken, rather it is a sliding scale of relevance to a country depending on its stage of economic development and absolute need. In short, countries that are trying to catch up with economic competitors may limit intellectual property rights, and history teaches us that some countries that today have sophisticated intellectual property rights and technologically advanced industries did not always respect the letter of intellectual property law.

**Table 1:** The four stages in the economic development of agrochemicals (modified from Mahoney^1^)

		Development of distribution systems		Development of R&D capability			
	Development of manufacturing	National	International	Private sector	Public sector	IP systems	Regulation
STAGE 1 Storage	Importation of finished goods or assembly of parts into finished products	Small domestic market	Very little distribution except as a TOLL manufacturer	Very little R&D	Very little R&D	Initial development allowing patents for local inventors but no interest from foreign investors	Very limited regulatory processes
STAGE 2 Limited inputs	Production on license or copy	Growing local market of increasing interest to foreign companies – import substitution	Growing companies learning how to establish export links	R&D to understand technology either to produce on license or copy	Development of university and independent research centres – capacity building	Interest growing among foreign investors – local inventors starting to file more patents	Limited services but without enforcement capabilities
STAGE 3 Inputs	Manufacture of domestically developed high‐technology products	Rapid growing domestic market of interest to foreign companies	Increasing exports that account for a growing share of GNP	Small‐scale advanced R&D effort capable of creating new products for domestic and export market	Vast acceleration of funding for R&D – development of major research centres linking with private sector	Advanced IP system but with certain limitations such as lack of enforcement	Advanced capabilities but not at highest level because of a lack of enforcement capabilities
STAGE 4 High‐value inputs	Highest capabilities to produce high‐technology agrochemicals	Highly profitable market in both the public and private sectors, generating profits to support in part advanced research	Global companies	Generous support for agricultural research from basic to applied – large investment by private companies including large agrochemical manufacturers		Sophisticated system of IP management operating according to the requirements of the TRIPS Agreement	Sophisticated agency overseeing regulatory approvals of agrochemicals – government oversees efficacy trials and production facilities and enforces regulations

## 4 EUROPEAN UNION (EU) REGISTRATION PROCESS

**Figure 1:**
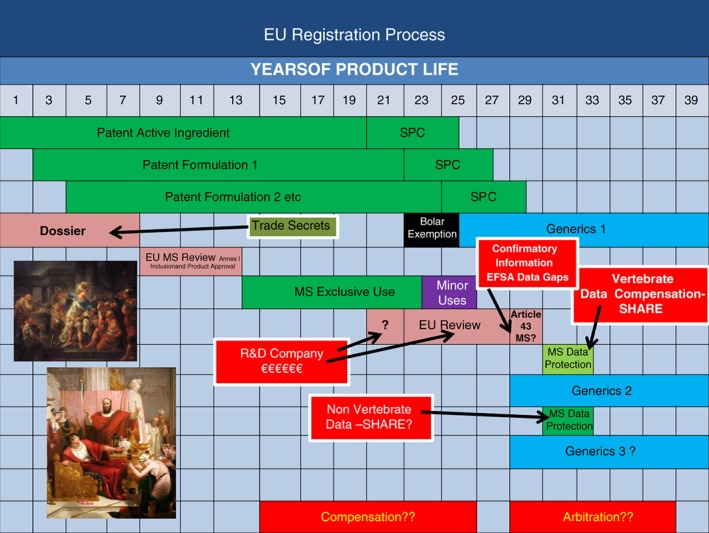
EU registration process for plant protection products.

Figure 1 shows a schematic representation of the EU registration process for chemical plant protection products. The 20 year patent protection can be supplemented by up to a further 5 years through use of supplementary protection certificates (SPCs) if market entry has been significantly delayed by the registration approval process.^2^ Once the chemical plant protection product is authorised at Member State (MS) level, 10 years of regulatory data protection or exclusive use is granted for that particular MS. A further 3 years maximum, exclusive use is possible if 12 minor uses are developed and authorised, which in this example goes past the patent expiry. The Bolar exemption^3^ allows companies other than the inventing company to develop their registration dossiers during the last couple of years of the patent or SPC, and eventually secondary manufacturers with generic versions of the original product enter the market. However, before patent expiry, the originating company must prepare for regulatory review, which is usually very expensive and takes an uncertain time as the exact requirements of the review are not specified by the regulator. From the regulator's point of view, this is a rational decision because not all parts of the regulatory risk assessment have reached scientific consensus and so it is difficult for the regulator to specify in advance what the review should consider. The problem for the registration holder is that they must effectively guess what the regulator may require, and this leads to overcompensation in that perhaps more studies are carried out than are eventually necessary. Not only is this expensive, it may lead to further complexity as studies may not resolve regulatory questions completely, leading to further studies and more expense. Generic registration holders may not have taken part in the review, and although this means they have not spent any money, there is great uncertainty about how their product authorisations will be dealt with at MS product review. However, task forces are formed in Europe between the primary and secondary manufacturers to defend active ingredients that have been off patent for several years. If the review is successful and the active substance is reapproved, then the data necessary for the regulatory decision will be protected for a period of 30 months from reregistration of the first product containing the active substance under review, when each MS reauthorises the product.^2^ The EU therefore gives serial regulatory data protection, first for 10 years and then for 30 months at each subsequent review. Vertebrate data necessary for the review regulatory decision must be shared for ethical reasons, with compensation payable to the data generator, but there is no forced sharing of non‐vertebrate data. This can lead to a lot of confusion and the possibility of duplication of studies and the resulting multiple data endpoints, which can cause chaos for the regulatory risk assessment.

## 5 US REGISTRATION PROCESS

**Figure 2:**
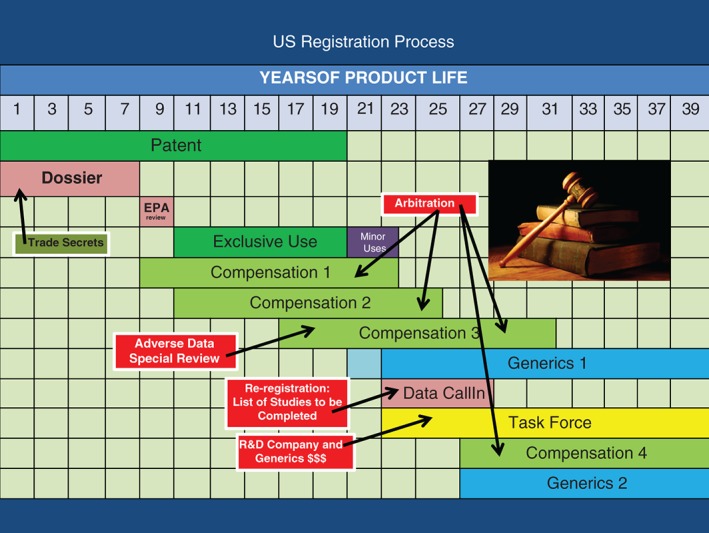
US registration process for plant protection products.

Figure 2 shows a schematic representation of the US registration process for chemical plant protection products. In contrast to the EU, there is no provision for supplementary patent protection, but the 10 year exclusive use period for regulatory data protection is the same as in the EU and starts at first registration. Minor use registrations can extend the exclusive use period in the United States in a similar fashion to the EU. In addition, all data on submission are compensable in that they have a value that must be paid by another party requiring access to the data. This period of compensation lasts for 15 years from data submission. Any data required by the US EPA or data that would change the risk assessment made by the US EPA are compensable and if submitted at different times will trigger separate compensable 15 year periods. There is no Bolar exemption in the United States, and so it is not possible to generate regulatory data towards the end of the patent period as is possible in the EU. Generic products eventually arrive after patent and exclusive use periods have expired and compensation has been paid. The review process^4^ in the United States is quite different from the EU, in that the US EPA specify what studies are required using a data call‐in (DCI) system. In its optimum form, the US EPA specify the studies required and the protocols to be followed, and give enough time for study completion. The registration holder may not like the final list of requirements, but at least there is some form of contract between regulator and registration holder. If there are multiple registration holders, acceptance of the review DCI allows those registration holders to stay on the market. Non‐compliance with the review DCI means that the registration holder will eventually have the approval cancelled. Multiple registration holders form task forces to share costs and avoid multiple studies for the same regulatory endpoint. Once data are submitted in the review to comply with the DCI, the data are compensable for 15 years from the date of submission. If further companies wish to enter the market post‐patent and after the regulatory data protection exclusive use period has expired, they must pay compensation for access. Therefore, the US EPA have a regulatory system that operates with a key insight, which is that, after the first 10 year period of regulatory data protection exclusive use, all further access to data is determined by ability to pay compensation. This avoids the relative complexity and uncertainty of the EU system, where sequential periods of regulatory data protection exclusive use detract from an efficient regulatory process. A final but important point is that, in order to obtain approvals on the basis of compensating data holders, the compensating company need only offer to pay. Thus, registrations can be granted and compensation may well end up being decided in the law courts through extended periods of mediation and arbitration.

## 6 CANADIAN REGISTRATION PROCESS

**Figure 3:**
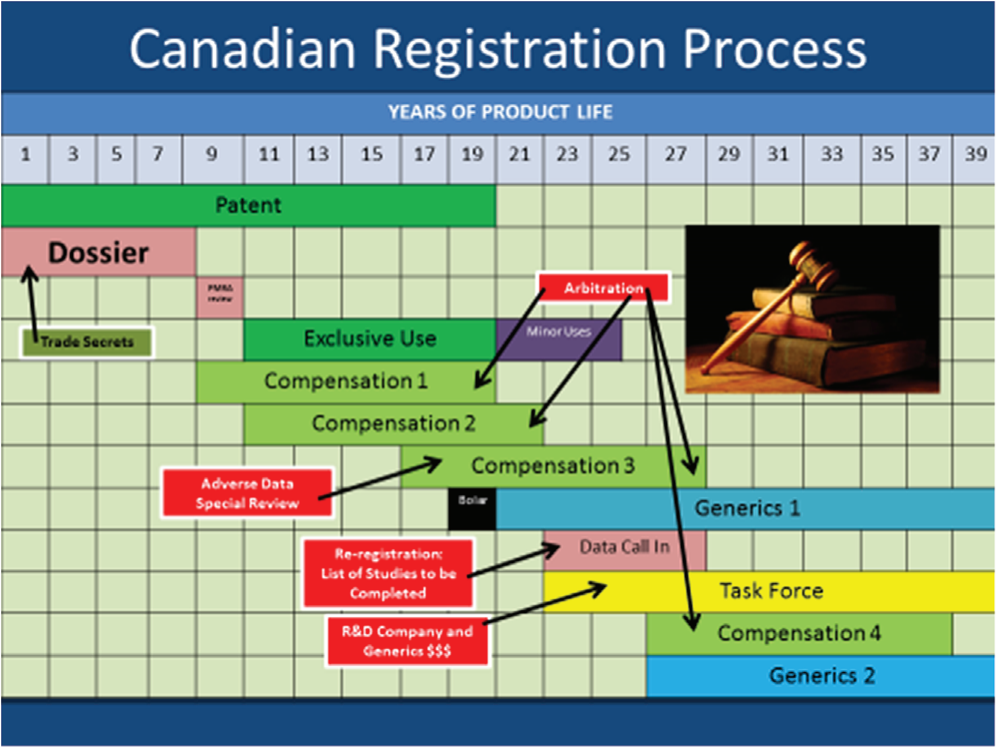
Canadian registration process for plant protection products.

Figure 3 shows a schematic representation of the Canadian registration process^5^ for chemical plant protection products. It is very similar to the US system, and regulatory review is based on DCI. The key differences are that there is a Bolar exemption and the compensation periods are only of 12 years duration. At the heart of the Canadian system is the key insight of the US EPA that, after the first 10 year period of regulatory data protection exclusive use, all further access to data is determined by ability to pay compensation, thus avoiding the complications of the EU process which utilises multiple sequential periods of regulatory data protection exclusive use. In Canada, however, it is not possible simply to offer to pay, and some form of compensation must be paid before a registration is granted, even if it is only the first of several instalments.

## 7 AUSTRALIAN REGISTRATION PROCESS

**Figure 4:**
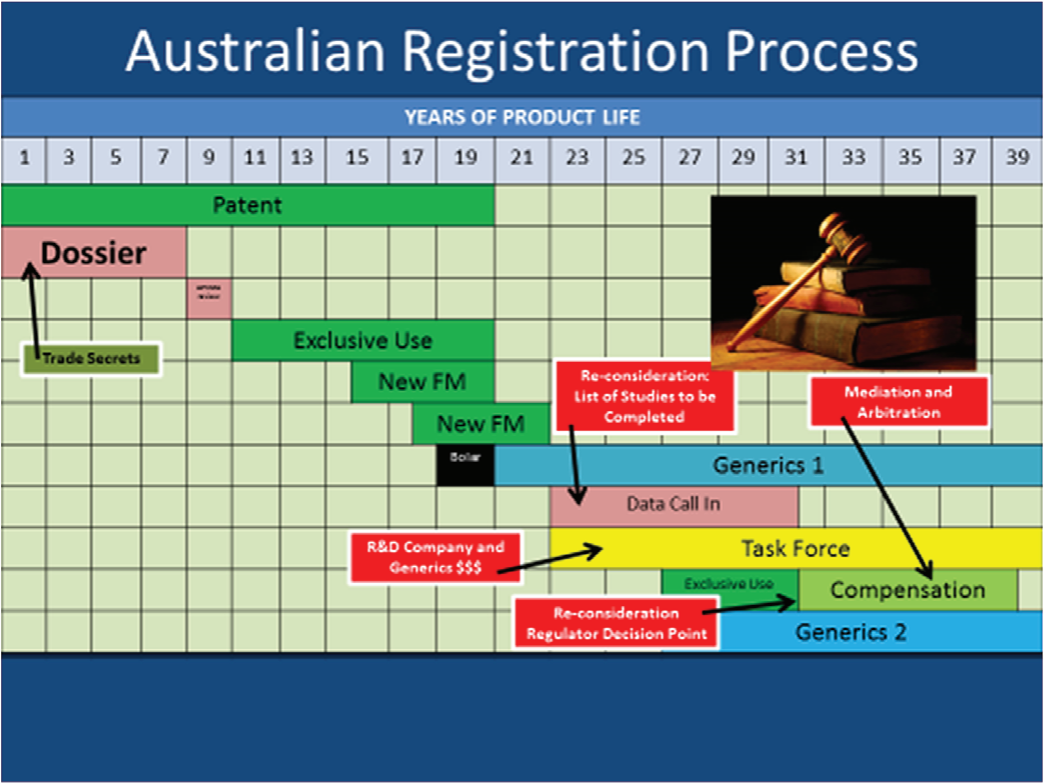
Australian registration process for plant protection products.

Figure 4 shows a schematic representation of the Australian registration process^6^ for chemical plant protection products. It is similar to the US and Canadian systems, but it does not have compensation at first registration. In addition, because the review of chemical plant protection products can take some time, data are given a limited protected period at submission in the review after a DCI process, and once the regulatory decision is taken to allow the registrations to continue, the protection is changed to 8 years of compensation. This is another example of quite an insightful adaptation because reviews always take much longer than expected and therefore any set period of compensation can be significantly eroded.

## 8 BRAZILIAN REGISTRATION PROCESS

**Figure 5:**
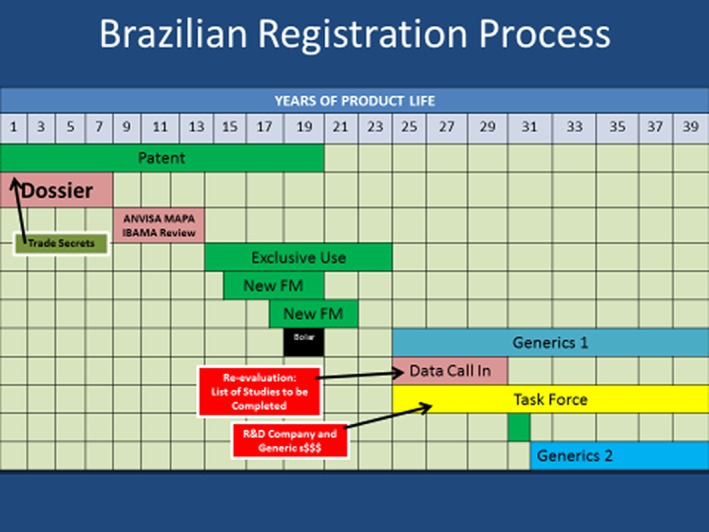
Brazilian registration process for plant protection products.

Figure 5 shows a schematic representation of the Brazilian registration process^7^ for chemical plant protection products. It is very similar to the Australian process, but instead of compensation at review a DCI process 1 year of regulatory data protection exclusive use is given.

## 9 CONCLUSIONS

Sophisticated intellectual property rights are the natural outcome of developed economies protecting their national company interests through time‐limited and perpetual rights to exploit novel inventions. In the specific case of chemical plant protection products, the regulatory process in many countries has added further intellectual property rights to those companies that can successfully demonstrate safety and efficacy by navigating extremely complex testing and risk assessment requirements. Eventually these rights are exhausted, and even perpetual rights such as trade secrets can be overcome indirectly, thus allowing generic products to compete with the original proprietary products. Healthy markets have a constant turnover of new and generic products that encourage novel inventions. The regulatory process may well be upsetting the delicate balance because the cost of demonstrating safety and efficacy of chemical plant protection products rises year on year, with no end in sight to these increasing costs.

The key insight of the US system is only to have one period of 10 years regulatory data protection exclusive use. Sequential periods of regulatory data protection exclusive use can lead to market disruption owing to overreliance on the regulatory process to limit market access to generic companies, which in the EU has led to a confused situation where data are generated at exceptional cost and yet compensation is not used to have a just distribution of the expense. Neither originating companies nor their generic competitors are happy because the regulatory system is extremely uncertain.

Of the five jurisdictions described (see Table 2 for a summary), four use a DCI system for review and three use a compensation system for generic access. The US, Canadian, Brazilian and Australian systems offer relatively rational regulatory processes, and the Australian system appears to be the most elegant in that compensation is only payable at review after first authorisation. DCI systems appear more rational and are used by four of the five jurisdictions described, but as scientific consensus on the regulatory utility of studies erodes at great speed, perhaps the EU regulators have predicted the future of regulatory systems for chemical plant protection products very well.

**Table 2:** Summary of the approach of different jurisdictions to regulatory data protection

Components	Canada	United States	European Union	Australia	Brazil
Protection period for a new active ingredient and associated formulated products	10 years exclusive use	10 years exclusive use	(*See notes re mandatory data sharing provisions*.) 10 years – plant protection products 13 years – low‐risk products 15 years – biocides (antimicrobials)	10 years (as of July 2014) (8 years prior to July 2014)	
Extensions linked to minor use registrations	Up to 5 additional years (1 year for each three minor uses)	Up to 3 additional years (1 year for each three minor uses)	Up to 3 additional years – plant protection products Up to 2 years – low‐risk products (3 months per minor use)	Up to 3 additional years (Prior to July 2014, 1 year for each five minor uses. Current provision not known)	
Total potential protection period	10–15 years	10–13 years	10–13 years – plant protection products 13–15 years – low‐risk protection products 15 years – biocides	10–13 years as of July 2014? (Prior to July 2014, 8–11 years)	
Data compensation period – subsequent data to support/maintain registration of a new active ingredient and to support re‐evaluation	12 years compensable	15 years compensable. Data with exclusive use protection are compensable for 5 years after exclusivity expires	30 months – plant protection products 5 years – review or renewal of biocides	5 years exclusive use 8 years – data for a reconsideration of a registration (as of July 2014)	1 year data protection for data call‐in studies
Notes			(EC 1107/2009 and 528/2012) Vertebrate test data are subject to compulsory sharing for both plant protection products and biocides. Applicants and resistrants are also encouraged to ‘make every effort’ to reach agreement on other data. Agreement on costs may be reached through negotiation, arbitration or legal proceedings	New legislation regarding agricultural chemicals (Ag Vet Code) took effect from July 2014. The full scope of the data protection provisions in the new Ag Vet Code is not apparent	
